# Do nitric oxide synthase and cyclooxygenase contribute to sweating response during passive heating in endurance‐trained athletes?

**DOI:** 10.14814/phy2.13403

**Published:** 2017-09-12

**Authors:** Tatsuro Amano, Naoto Fujii, Glen P. Kenny, Yoshimitsu Inoue, Narihiko Kondo

**Affiliations:** ^1^ Laboratory for Exercise and Environmental Physiology Faculty of Education Niigata University Niigata Japan; ^2^ Institute of Health and Sport Sciences University of Tsukuba Tsukuba Japan; ^3^ Human and Environmental Physiology Research Unit University of Ottawa Ottawa Canada; ^4^ Laboratory for Human Performance Research Osaka International University Osaka Japan; ^5^ Laboratory for Applied Human Physiology Graduate School of Human Development and Environment Kobe University Kobe Japan

**Keywords:** Eccrine sweat glands, exercise training, heat acclimation, prostaglandins, thermoregulation

## Abstract

The aim of our study was to determine if habitual endurance training can influence the relative contribution of nitric oxide synthase (NOS) and cyclooxygenase (COX) in the regulation of sweating during a passive heat stress in young adults. Ten trained athletes and nine untrained counterparts were passively heated until oral temperature (as estimated by sublingual temperature, T_or_) increased by 1.5°C above baseline resting. Forearm sweat rate (ventilated capsule) was measured at three skin sites continuously perfused with either lactated Ringer's solution (Control), 10 mmol/L *N*^*G*^‐nitro‐_L_‐arginine methyl ester (_L_‐NAME, non‐selective NOS inhibitor), or 10 mmol/L ketorolac (Ketorolac, non‐selective COX inhibitor) via intradermal microdialysis. Sweat rate was averaged for each 0.3°C increase in T_or_. Sweat rate at the _L_‐NAME site was lower than Control following a 0.9 and 1.2°C increase in T_or_ in both groups (all *P *≤* *0.05). Relative to the Control site, NOS‐inhibition reduced sweating similarly between the groups (*P *=* *0.51). Sweat rate at the Ketorolac site was not different from the Control at any levels of T_or_ in both groups (*P *>* *0.05). Nevertheless, a greater sweat rate was measured at the end of heating in the trained as compared to the untrained individuals (*P *≤* *0.05). We show that NOS contributes similarly to sweating in both trained and untrained individuals during a passive heat stress. Further, no effect of COX on sweating was measured for either group. The greater sweat production observed in endurance‐trained athletes is likely mediated by factors other than NOS‐ and COX‐dependent mechanisms.

## Introduction

It is well‐established that endurance trained individuals show an enhanced sweat production relative to their untrained counterparts during a passive (Amano et al. 2013; Tokizawa et al. 2016) or exercise‐induced (Piwonka et al. 1965; Yamazaki et al. 1994; Ichinose‐Kuwahara et al. 2010) heat stresses. To date the mechanisms governing the elevated sweating response in endurance‐trained athletes remains unclear. It is possible, however, that the greater sweat production may in part be due to the hypertrophy of eccrine sweat glands (Sato and Sato 1983) and or enhanced cholinergic responsiveness of the sweat glands (Buono and Sjoholm 1988; Wilson et al. 2010; Amano et al. 2013; Inoue et al. 2014) associated with endurance training. A growing body of evidence has shown that nitric oxide (NO) (Lee and Mack 2006; Welch et al. 2009; Fujii et al. 2014b, 2015, 2016b; Stapleton et al. 2014; Louie et al. 2016) and cyclooxygenase (COX) (Sato 2001; Fujii et al. 2014b) mediates the regulation of sweating and may therefore play a role in governing the endurance‐training induced elevation in sweating.

A number of investigations have observed an attenuation of sweating with the intradermal administration of a NO synthase (NOS) inhibitor during exercise (Welch et al. 2009; Fujii et al. 2014b, 2015, 2016b; Stapleton et al. 2014; Louie et al. 2016). Moreover, we recently showed that individuals who exhibited a higher sweat rate during exercise demonstrated a greater NOS‐dependent sweating (Amano et al. 2017) which lends support to the hypothesis that NOS may play an integral role in mediating the greater sweat production typically observed in endurance trained individuals (Yamazaki et al. 1994; Ichinose‐Kuwahara et al. 2010; Amano et al. 2011, 2013; Tokizawa et al. 2016). Additionally, recent studies showed that COX inhibition attenuates sweat rate during exercise (Fujii et al. 2014b, 2016a). Noteworthy, increases in prostaglandin production via the COX pathway have been shown to induce increases in sweat production in vitro (Sato 2001) albeit others have reported no influence of COX or prostaglandins (Charkoudian and Johnson 1999; Bradford et al. 2007; Fujii et al. 2014a, 2016c; Haqani et al. 2017). While the underlying reasons for this disparity remain unclear, our recent study showed that NOS‐ (Fujii et al. 2015; Amano et al. 2017) and COX‐ (Fujii et al. 2015) dependent sweating is variable between individuals. Given that the COX‐ and NOS‐dependent modulation of sweating may occur via a similar pathway (Fujii et al. 2014b), the lack of an observed effect of COX in the modulation of sweating may be related to individual variations as observed with NOS. Taken together, it is plausible therefore that in addition to NOS, COX may contribute to the greater sweat production observed in endurance‐trained individuals. However, the relative contribution of each modulator remains to be established.

Thus, the purpose of present study was to investigate the relative contribution of NOS and COX on the sweating response in young untrained and endurance‐trained adults during a heat stress. We hypothesized that while the inhibition of NOS and COX would attenuate sweat rate during a passively‐induced increase in body core temperature in both groups, the magnitude of the reduction would be greater in endurance‐trained as compared to the untrained individuals.

## Materials and Methods

### Ethical approval

The present study was approved by a human ethical committee of Faculty of Education, Niigata University with declare of the latest version of Helsinki. Verbal and written informed consent was obtained from all participation.

### Participants

Young endurance‐trained (*n* = 10, 8 males and 2 females) and untrained adults (*n* = 9, 6 males and 3 females) participated in the study. The endurance‐trained individuals were competitive distance runners who were members of the athletic club at Niigata University and were engaged in endurance running training at least five times per week for at least 3 years. Their best competitive 5000 m times were 15.44 ± 0.22 min and 21.07 ± 1.31 min for the males (*n* = 8) and females (*n* = 2), respectively. It has previously been reported that long‐ and middle‐distance runners with similar training backgrounds show a greater sweating response relative to the untrained counterparts during a passively induced heat stress (Amano et al. 2013; Tokizawa et al. 2016). The untrained participants had not been engaged in regular exercise training for at least 3 years. All female subjects participated in the experiment during the early follicular phase (within 6 days of starting menstruation). None of the female subjects reported using contraceptives prior to their participation in the study.

### Experimental protocol

All experimental trials were conducted in a thermoneutral environment (25.0 ± 0.2°C). Upon arrival to the laboratory, the participant's body weight was measured. They subsequently donned a water perfusion suit (Allen‐Vanguard, Ottawa, Canada) under which they wore a cotton T‐shirt. The participants then rested in a semirecumbent position during which time skin temperature was clamped at 33–34°C by circulating water regulated at 34°C. During this time, using aseptic techniques, three microdialysis fibers were inserted into the dermal layer of skin on the dorsal side of the left forearm. A 25‐gauge needle was first introduced into the unanesthetized skin and exited ~2.0 cm distally from the insertion point. The microdialysis fiber (50 kDa cut‐off, 10 mm membrane) (EIM‐580; Eicom, Kyoto, Japan) was then threaded through the lumen of the needle, which was subsequently withdrawn leaving the fibre in place. Each of the fibers were separated by at least 2.0 cm. Ten minutes after the last fibre insertion, the microdialysis probes were perfused with (1) lactated Ringer's solution (Control, Terumo, Tokyo, Japan), (2) 10 mmol/L *N* ^G^‐nitro‐arginine methyl ester, a non‐selective NOS inhibitor (_L_‐NAME, Acros Organic, NJ), and (3) 10 mmol/L ketorolac, a non‐selective COX inhibitor (Ketorolac, Tokyo chemical industry, Tokyo, Japan) in a counter‐balanced manner. The infusion rate was maintained at 4 *μ*l/min via a micro‐infusion pump (YSP‐101; Ymc, Kyoto, Japan). The concentrations of _L_‐NAME and ketorolac employed were based on previous studies using a comparable technique (Minson et al. 2001; Holowatz et al. 2005; Kellogg et al. 2005; Shibasaki et al. 2007; Wong 2013; Fujii et al. 2014b). Each drug was perfused for a minimum of 75 min prior to the start of the experimental protocol to establish the required blockade. Combined with the pre‐infusion baseline resting period, a total of 85 min had elapsed prior to the start of the experiment. This delay period has been shown to be sufficient to ensure that any trauma associated with the insertion of the probe had subsided (Hodges et al. 2009).

After a 5‐min baseline data collection period, whole‐body heating was initiated by circulating water regulated at 44°C through the perfusion suit and simultaneously immersing the lower legs in warm water maintained at 41°C. Thereafter, the water temperature perfusing the suit was increased by 2°C increments every 10 min to a maximum of 52°C which was maintained for the duration of the heating protocol. Ten minutes after the initiation of heating, the temperature of the lower leg water bath was increased to 43°C and was maintained at this temperature for the duration of the trial. The heating protocol was continued until oral temperature (T_or_) increased by 1.5°C above the pre‐heating baseline resting levels.

### Measurements

Sublingual temperature using a general purpose type‐T thermocouple temperature probe (Inui Engineering, Higashi Oosaka, Japan) was used as an estimate of T_or_. Participants were asked to breathe through their nose only during the measurement of sublingual temperature. Skin temperatures were measured at seven skin sites using thermocouples affixed to the skin surface with surgical tape. Mean skin temperature was calculated using the 7 skin temperatures weighted to the regional proportions determined as follows (Mitchell and Wyndham 1969): forehead 7%, abdomen 35%, forearm 14%, hand 5%, thigh 19%, lower leg 13%, and foot 7%.

Local sweat rate was measured continuously using the ventilated capsule method. A 1.0 cm^2^ plastic capsule was affixed at each site using adhesive tapes and topical glue (Collodion; Kanto chemical, Tokyo, Japan). The participant was required to rest their forearm on a table maintained at heart level throughout the experiment. Dry nitrogen gas was passed through each capsule and over the skin surface at a rate of 0.8 L/min. Water content from the effluent air was measured using a capacitance hygrometer (HMP60; Vaisala, Helsinki, Finland). All temperature and sweat rate data were recorded at 1‐sec intervals using a data logger (MX100; Yokogawa, Tokyo, Japan) and simultaneously displayed on a computer screen (MX100 standard software). Heart rate (HR) was monitored continuously using a telemetry transmitter strapped around the chest (RS800; Polar Electro Oy, Kempele, Finland).

### Data and statistical analyses

Baseline variables were averaged during the 5 min resting period. During the heating phase, all variables were averaged at 0.3°C increments in T_or_. Mean body temperature was calculated as 0.8 × T_or_  + 0.2 × mean skin temperature (Stolwijk and Hardy 1966). NOS inhibition attenuated sweat rate when the T_or_ was elevated to 0.9 and 1.2°C above baseline (see results). Thus, we averaged sweat rate at these two levels of T_or_ to evaluate NOS‐ or COX‐dependent sweating as assessed by the differences in sweat rate between the _L_‐NAME or Ketorolac sites relative to the Control site. Responses were compared between groups. In addition, NOS‐ or COX‐dependent sweating was plotted against sweat rate measured at the Control site for each group in order to evaluate whether levels of sweat rate relates to NOS‐ or COX‐dependent sweating.

Sweat rate was analyzed using a three‐way mixed‐design ANOVA with factors of treatment site (three levels: Control, _L_‐NAME, and Ketorolac), level of increase in T_or_ above baseline resting levels (six levels: baseline, 0.3, 0.6, 0.9, 1.2, and 1.5°C), and group (two levels: endurance‐trained and untrained). HR, T_or_, mean skin temperature, and mean body temperature were analysed using a two way mixed‐design ANOVA with factors of T_or_ (six levels: baseline, 0.3, 0.6, 0.9, 1.2, and 1.5°C) and group. When a significant main effect or an interaction was observed, a post hoc for multiple comparison was conducted with a Student's pairwise or nonpairwise *t*‐test. Sweat rates at _L_‐NAME and Ketorolac sites were compared to the Control sites for the within treatment sites analysis. *P* values for the multiple comparisons were adjusted using the Bonferroni's procedure. A Student's nonpairwise *t*‐test was employed to compare NOS‐ or COX‐dependent sweating between groups. Linear regression analysis was performed to determine the relationship between individual changes in sweat rate at the _L_‐NAME or Ketorolac sites from the Control site and the individual sweat rate achieved at the Control site. The participant's characteristics (i.e., age, height, weight, and body surface area) were compared between the groups by a Student's non‐pairwise *t*‐test. Data were presented as means ± SD, and statistical significance was set at *P *≤* *0.05. All statistical analyses were performed using a statistical package (SPSS) version 24.0.

## Results

Age (20 ± 1 and 21 ± 3 years), height (1.68 ± 0.08 and 1.71 ± 0.07 m), weight (59 ± 5 and 59 ± 6 kg), and body surface area (1.66 ± 0.12 and 1.69 ± 0.10 m^2^) were similar between untrained and trained groups, respectively (all *P *>* *0.05). No differences in heating time were measured between groups (54 ± 8 and 53 ± 9 min for the untrained and endurance‐trained groups, respectively, *P *=* *0.98). A main effect of group was observed on HR during passive heating such that a lower HR was measured in the endurance‐trained relative to the untrained group (*P *<* *0.01, Table 1). T_or_, mean skin temperature, and mean body temperature during passive heating were not different between the groups (all *P *>* *0.05).

**Table 1:** Physiological variables during passive heating in untrained individuals (*n* = 9) and trained athletes (*n* = 10)

	Changes in oral temperature from baseline resting (°C)					
	BL	0.3	0.6	0.9	1.2	1.5
HR (beats min^−1^)^a^						
Untrained	77 (20)	95 (16)	105 (18)	116 (19)	126 (20)	132 (21)
Trained	53 (7)	65 (4)	74 (5)	82 (7)	89 (9)	93 (10)
Oral temperature (C°)						
Untrained	36.91 (0.31)	37.16 (0.30)	37.46 (0.31)	37.76 (0.31)	38.06 (0.31)	38.30 (0.30)
Trained	36.82 (0.28)	37.06 (0.27)	37.36 (0.27)	37.65 (0.27)	37.96 (0.27)	38.20 (0.28)
Mean skin temperature (C°)						
Untrained	33.33 (1.07)	36.58 (0.98)	36.99 (0.76)	37.38 (0.64)	37.75 (0.59)	37.95 (0.59)
Trained	33.59 (0.39)	36.86 (0.34)	37.17 (0.39)	37.51 (0.42)	37.82 (0.40)	38.04 (0.43)
Mean body temperature (C°)						
Untrained	36.14 (0.41)	37.05 (0.39)	37.36 (0.36)	37.69 (0.35)	38.00 (0.35)	38.24 (0.33)
Trained	36.08 (0.25)	37.02 (0.23)	37.32 (0.24)	37.62 (0.26)	37.93 (0.26)	38.17 (0.28)

Values given are the means (SD). BL, baseline. HR, heart rate.

a Significant main effect of group (*P < *0.01).

**Figure 1:**
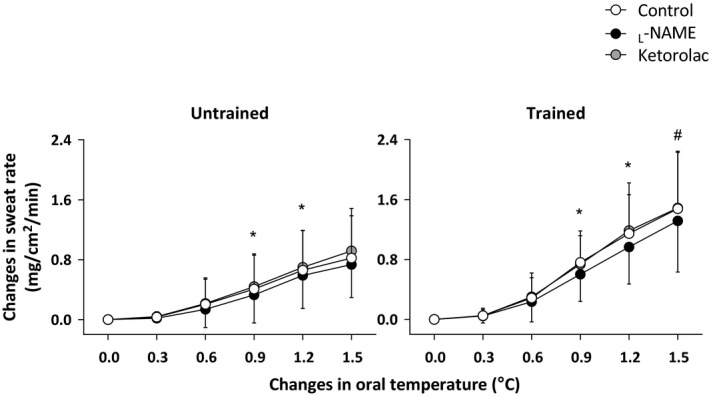
Changes in sweat rate during passive heating in the untrained (*n* = 9) and endurance‐trained (*n* = 10) participants. Data were presented as mean ± SD. *, between the Control and _L_‐NAME sites (*P *≤* *0.05). #, versus untrained (*P *≤* *0.05).

Figure 1 shows the sweating responses at all treatment sites as a function of increasing T_or_. A significant interaction was observed between T_or_ and group such that sweat rate was higher in the endurance‐trained group relative to their untrained counterparts at a 1.5°C increase in T_or_ (*P *≤* *0.05, Fig. 1). Similarly, a significant interaction of treatment site and T_or_ was measured whereby sweat rate at the _L_‐NAME site was lower than the Control at a 0.9 and 1.2°C elevation in T_or_ whereas the Ketorolac and Control sites were not different at any levels of T_or_ (all *P *>* *0.05, Fig. 1).

**Figure 2:**
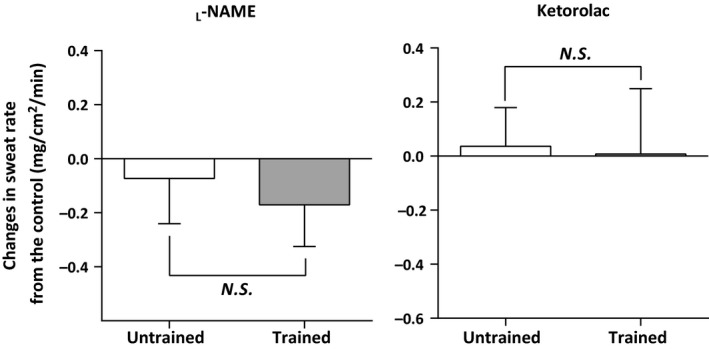
Group comparison of the changes in sweat rate at the _L_‐NAME and Ketorolac sites relative to the Control site at the end of passive heating. Data were presented as mean ± SD for *n* = 10 and 9 in endurance‐trained and untrained groups, respectively.

Figure 2 demonstrates the between‐group comparisons for the change in sweat rate at both the _L_‐NAME and Ketorolac sites relative to the Control site, at a 0.9 and 1.2°C increase in T_or_ where the attenuation of sweat rate at the _L_‐NAME site was evident. No group differences in the change in sweat rate for both treatment sites (i.e., _L_‐NAME and Ketorolac) from the Control site were observed (all *P *>* *0.05, Fig. 2).

**Figure 3:**
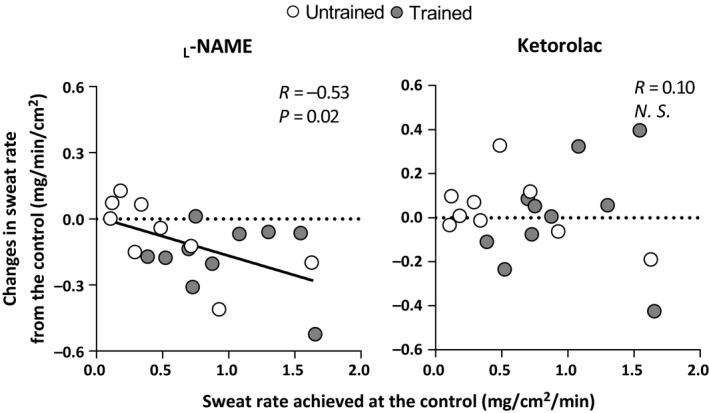
Individual changes in sweat rate at the _L_‐NAME or Ketorolac sites relative to the Control site as a function of sweat rate achieved at the Control during passive heating (*n* = 19 in total).

Figure 3 shows the individual changes in sweat rate at both the _L_‐NAME and Ketorolac sites from the Control site as a function of the sweat rate achieved at the Control at a 0.9 and 1.2°C increase in T_or_. The change in sweat rate at the _L_‐NAME site from Control was negatively correlated with the levels of sweat rate at the Control site (*P *=* *0.02, *R* = −0.53, Fig. 3). In contrast, no association was observed at the Ketorolac site (*P *=* *0.67, *R* = −0.10, Fig. 3).

## Discussion

To our knowledge, this is the first study to evaluate the relative contribution of NOS and COX in the regulation of the sweating in endurance‐trained athletes during a passive heat stress. We showed that NOS inhibition similarly attenuated sweat rate relative to the Control site in both untrained and endurance‐trained individuals following T_or_ increments of 0.9 and 1.2°C above resting levels. In contrast, this attenuation was abolished at the highest level of hyperthermia achieved (i.e., T_or_ increase in 1.5°C) in both groups. However, contrary to our hypothesis, the magnitude of this NOS‐mediated reduction in sweating was not different between groups. Furthermore, we showed that relative to the Control site, COX inhibition did not influence sweat rate in either group or for any level of heat stress. These results suggest that endurance training does not affect the relative contribution of NOS to the sweating response which occurred at moderate‐to‐high levels of hyperthermia only in young adults. Moreover, we show that COX is not involved in the observed endurance‐training‐induced increases in sweat production.

### NOS‐dependent sweating

It is well‐recognized that NOS contributes to the sweating response during exercise in young adults (Lee and Mack 2006; Welch et al. 2009; Fujii et al. 2014b, 2015, 2016b; Stapleton et al. 2014; Louie et al. 2016). However, until the present study, the influence of training status on NOS‐dependent sweating had not been considered. Consistent with previous reports (Amano et al. 2013; Tokizawa et al. 2016), our young endurance‐trained individuals showed a greater sweating response compared with their untrained counterparts at the end of passive heating (Fig. 1). Interestingly, we showed an attenuation in sweating with the inhibition of NOS at moderate‐to‐high levels of hyperthermia equivalent to a 0.9 and 1.2°C increase in T_or_ only in both groups (Fig. 1) and the magnitude of attenuation was similar between groups (Fig. 2). Furthermore, while we observed a higher sweat rate in the endurance‐trained individuals relative to the untrained counterparts, it was most evident at the end of passive heating when the contribution of NOS to sweating response in both groups was absent (Fig. 1). These results suggest that endurance training per se does not modulate NOS‐dependent sweating during passive heating. That is, endurance training appears to improve the body's ability to sweat independently of NOS.

Interestingly however, we observed a relationship between NOS‐dependent sweating and the levels of sweat rate at the Control site (Fig. 3). We recently observed a similar relationship during moderate intensity exercise in the heat in a larger cohort of participants of varying levels of fitness (*n* = 46; range of *V*O_2peak_, 28.0–62.3 mLO_2_ kg^−1^ min^−1^) (Amano et al. 2017). These findings suggest that NOS‐dependent sweating is most evident in individuals who demonstrate greater sweat output regardless of their training status. Noteworthy, in the present study we observed a coefficient of determination (*R* ^2^) of 0.28 in the relationship between NOS‐dependent sweating and sweat rate at the Control site (Fig. 3). This suggests that the contribution of NOS to the individual variation of sweat production is approximately 30% during passive heating which is in accordance with our previous observations of a similar contribution of NOS‐dependent sweating in exercise (*R* ^2^ = 0.19–0.26) (Amano et al. 2017). Therefore, while NOS partly (e.g., ~30%) contributes to individual variations of sweat production, this contribution does not appear to account for the higher sweat production measured in our endurance‐trained individuals as compared to their untrained counterparts. In keeping with this observation, previous studies have demonstrated that the size of eccrine sweat glands (Sato and Sato 1983) and the cholinergic responsiveness of the sweat glands (Sato and Sato 1983; Buono and Sjoholm 1988; Wilson et al. 2010; Amano et al. 2013; Inoue et al. 2014) are also important determinants of sweat production in physically trained adults. Whether these factors contributed to the group differences in sweat production in the present study requires further scrutiny.

Previous studies have reported a NOS‐dependent sweating during moderate (Welch et al. 2009; Fujii et al. 2014b, 2015, 2016b; Stapleton et al. 2014; Louie et al. 2016) but not high (Fujii et al. 2014b) intensity exercise when the requirements for sweating were greater. Consistent with these observations, we observed a NOS‐dependent sweating at moderate‐to‐high increases in oral temperature of between 0.9 and 1.2°C above baseline resting levels only. This response was absent at the highest increase in oral temperature of 1.5°C in both groups where sweat production was the greatest (Fig. 1). The underlying mechanism for this latter response cannot be elucidate from the current study. However, it has been shown that intradermally administered methacholine‐induced sweating response was attenuated by _L_‐NAME at low doses of methacholine but not higher doses (Lee and Mack 2006). Given that the elevated core temperature may promote greater acetylcholine release from sympathetic nerve ending (Shibasaki et al. 2013), it is plausible that the levels of acetylcholine in the skin may contribute to abolishment of the NOS‐dependent sweating response at high elevated levels of hyperthermia (i.e., >1.2°C) as observed at the end of passive heating in the present study. Further studies are required to elucidate the extent to which NOS mediates the regulation of sweating as a function of different levels of hyperthermia during a passive heat stress.

Inconsistent with the present observation, it was reported that NOS inhibition does not modulate sweating response at a similar elevations in core temperature (i.e., 1.0°C) during passive heating (Haqani et al. 2017). While the underlying reason(s) for this discrepancy is unknown, it is assumed that individual differences in the contribution of NOS to sweating may in part be a factor. Further studies are required to elucidate the precise mechanisms of the individual variation in NOS on the contribution of sweating during passive heating as a function of different levels of hyperthermia.

### COX‐dependent sweating

Recent studies have reported divergent effects of COX inhibition on the sweating response in humans in vivo (Fujii et al. 2014a,b, 2015, 2016a; Haqani et al. 2017). For example, COX inhibition has been shown to attenuate sweat rate during exercise in the heat (Fujii et al. 2014b, 2015, 2016a) but not during resting under either normothermic (Fujii et al. 2014a) or hyperthermic (1.0°C elevation of rectal temperature) (Haqani et al. 2017) conditions. Consistent with the recent study under resting hyperthermic conditions in non‐endurance trained young males (i.e., exercised for no more than 2–3 times per week for 30 min duration) (Haqani et al. 2017), we observed that COX inhibition does not alter sweat rate during passive heating in untrained individuals and extend upon this observation by demonstrating a similar response in endurance‐trained individuals (Fig. 1). These results suggest that the observed differences in sweat production between trained and untrained individuals is independent of COX‐related mechanisms. Unlike the sweating response at the _L_‐NAME site, we did not observe a relationship between the COX‐dependent sweating and the magnitude of sweating at the Control site (Fig. 3). This infers that individuals who have a high sweat capacity do not necessarily exhibit greater COX‐dependent sweating. Further studies are required to extend our understanding of the mechanisms underpinning COX‐dependent sweating during passive heating.

### Perspectives and Significance

It is well recognized that endurance‐trained athletes have a greater sweating response during an exercise‐ and passively induced heat stress relative to their untrained counterparts (Piwonka et al. 1965; Baum et al. 1976; Yamazaki et al. 1994, 1996; Yanagimoto et al. 2002; Ichinose‐Kuwahara et al. 2010; Amano et al. 2011, 2013; Tokizawa et al. 2016). This response has been ascribed to sweat gland hypertrophy (Sato and Sato 1983) and or higher cholinergic responsiveness following exercise training or heat acclimation (Buono and Sjoholm 1988; Wilson et al. 2010; Amano et al. 2013; Inoue et al. 2014). However, the underlying mechanism(s) associated of sweat gland adaptation to exercise training remains incomplete. The present study provides important new insights into our understandings of the mechanism(s) associated with as it relates to the relative contribution of NOS‐ and COX‐dependent mechanisms.

### Limitations

We did not measure *V*O_2peak_ in the present study. As such, differences in fitness between trained and untrained individuals cannot be determined and its influence on our study findings cannot be elucidated. However, studies show that differences in aerobic power (as defined by level of *V*O_2peak_) is not necessarily associated with improvements in sweat production (Amano et al. 2013, 2017). Thus, while we are unable to determine the possible influence of fitness on our study findings, based on prior evidence, it is unlikely to have a significant influence.

In conclusion, we show that NOS‐dependent sweat production during passive heating does not differ between young endurance‐trained and untrained individuals. In addition, we show that irrespective of the individuals training status, COX does not mediate changes in sweating during progressive increase in core temperature associated with passive heating. Finally, our findings demonstrate that the greater sweat production observed in endurance‐trained athletes is likely mediated by factors other than NOS‐ and COX‐dependent mechanisms.

## Conflicts of Interest

None declared.
